# High body mass index is not associated with atopy in schoolchildren living in rural and urban areas of Ghana

**DOI:** 10.1186/1471-2458-11-469

**Published:** 2011-06-14

**Authors:** Irene A Larbi, Kerstin Klipstein-Grobusch, Abena S Amoah, Benedicta B Obeng, Michael D Wilson, Maria Yazdanbakhsh, Daniel A Boakye

**Affiliations:** 1 Division of Epidemiology & Biostatistics, School of Public Health, Faculty of Health Sciences, University of the Witwatersrand, Johannesburg, South Africa; 2 Department of Parasitology, Noguchi Memorial Institute for Medical Research, University of Ghana, Legon-Accra, Ghana; 3 Julius Center for Health Sciences and Primary Care, Utrecht University Medical Center, Utrecht, The Netherlands; 4 Department of Parasitology, Leiden University Medical Centre, Leiden, The Netherlands

## Abstract

**Background:**

Factors which determine the development of atopy and the observed rural-urban gradient in its prevalence are not fully understood. High body mass index (BMI) has been associated with asthma and potentially atopy in industrialized countries. In developing countries, the transition from rural to urban areas has been associated with lifestyle changes and an increased prevalence of high BMI; however, the effect of high BMI on atopy remains unknown in this population. We therefore investigated the association between high BMI and atopy among schoolchildren living in rural and urban areas of Ghana.

**Methods:**

Data on skin prick testing, anthropometric, parasitological, demographic and lifestyle information for 1,482 schoolchildren aged 6-15 years was collected. Atopy was defined as sensitization to at least one tested allergen whilst the Centres for Disease Control and Prevention (CDC, Atlanta) growth reference charts were used in defining high BMI as BMI ≥ the 85^th ^percentile. Logistic regression was performed to investigate the association between high BMI and atopy whilst adjusting for potential confounding factors.

**Results:**

The following prevalences were observed for high BMI [Rural: 16%, Urban: 10.8%, p < 0.001] and atopy [Rural: 25.1%, Urban: 17.8%, p < 0.001]. High BMI was not associated with atopy; but an inverse association was observed between underweight and atopy [OR: 0.57, 95% CI: 0.33-0.99]. Significant associations were also observed with male sex [Rural: OR: 1.49, 95% CI: 1.06-2.08; Urban: OR: 1.90, 95% CI: 1.30-2.79], and in the urban site with older age [OR: 1.76, 95% CI: 1.00-3.07], family history of asthma [OR: 1.58, 95% CI: 1.01-2.47] and occupational status of parent [OR: 0.33, 95% CI: 0.12-0.93]; whilst co-infection with intestinal parasites [OR: 2.47, 95% CI: 1.01-6.04] was associated with atopy in the rural site. After multivariate adjustment, male sex, older age and family history of asthma remained significant.

**Conclusions:**

In Ghanaian schoolchildren, high BMI was not associated with atopy. Further studies are warranted to clarify the relationship between body weight and atopy in children subjected to rapid life-style changes associated with urbanization of their environments.

## Background

Allergic diseases are among the most prevalent chronic conditions in pediatric populations [1]. Although, the high infectious disease burden in Africa has resulted in the setting of health priorities around human immunodeficiency virus, tuberculosis and malaria; recent studies indicate that childhood allergies are an emerging health problem for Africa [2,3]. Moreover, the observed rural-urban gradient in the prevalence of allergies [2-5] has raised concerns as to whether the high prevalence in urban areas is due to environmental changes or if urban populations are just becoming prone to allergies. Though there is a paucity of literature on the effect of migration within non-industrialized countries on allergies, findings of international migrant studies indicate that migration from developing to industrialized countries is associated with an increased prevalence of allergies [6].

In view of the fact that the African continent is currently experiencing a rapid drift in rural-urban migration and the fact that children under the age of 15 years, who are worst affected by allergies, constitute about 50 percent of the population in developing countries, [5] it is crucial to understand the factors which determine the rural-urban gradient in the prevalence of allergies. Atopy, the genetic predisposition of some individuals to develop immunoglobulin E antibodies (IgE) in response to allergen exposure [7], normally precedes the development of allergies [8] and accounts for about 50 percent of all cases of allergies [9-12]. The factors which influence the development of atopy are however not fully understood [13].

High body mass index (BMI) has been implicated in the development of asthma in industrialized countries [14] however, the relationship between high BMI and atopy is not clearly established [15], but potentially mediated through elevated production of leptin, tumor necrosis factor α (TNFα) and interleukin-6 associated with increased adiposity. High levels of TNFα cause an increase in the production of T-helper lymphocyte type 2 (Th2) cytokines such as interleukin-4 and interleukin-5; which are primary signals for activating an immune response towards atopy [15].

Like in many developing economies in the world, over-nutrition exist side-by-side with under-nutrition in the Ghanaian population. Though statistics are not readily available for children of school-going age in Ghana, records for children under the age of five indicate that the prevalence of overweight increased by 3.8 folds (from 0.5% to 1.9%) between 1988 and 1994 [16]. As of 2008, the national prevalence of overweight was 5% in children under the age of five [17].

Previous studies have reported that the prevalence of childhood atopy is usually low in rural areas in Africa where children are normally shorter and lighter than their urban counterparts [3,18,19] however; the effect of high BMI, which is increasingly becoming more prevalent in developing countries [20,21] on atopy has barely been explored in rural and urban areas in Africa. The current study sought to explore the effect of high BMI on atopy among school children living in rural and urban areas of Ghana.

## Methods

### Study sites

**Figure 1:**
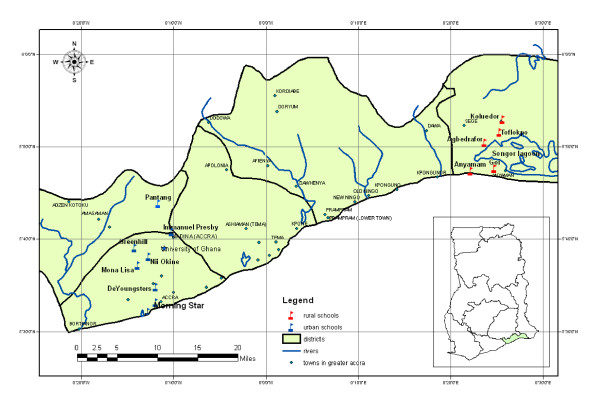
**Map of the Greater Accra Region showing locations of the recruited rural and urban schools**.

This study was conducted in 2006 in three districts in the Greater Accra Region of Ghana; two urban (Accra Metropolitan Area and Ga) and one rural district (Dangme East) (see Figure 1). Compared to rural settlements, urban areas are more developed in terms of basic infrastructure and lifestyle.

### Study population

The study population comprised children attending mixed day basic-level schools in the three selected districts who were in the age range of 6-15 years. The recruited schools in the rural site were public whilst those in the urban site were mainly public and middle-class private schools. The response rate for the study was 59% [urban site: 54% (1111/2064); rural site: 68% (815/1200)]. Parents or guardians consented to their children's participation in the study by signing or thumb printing a consent form. Ethical approval for the study was granted by the Institutional Review Board of the Noguchi Memorial Institute for Medical Research in Accra and the Human Research Ethics Committee of the University of the Witwatersrand, Johannesburg.

### Measurements

#### Atopy

Atopy was assessed by skin prick testing (SPT) on the volar forearms of each participant with a panel of standardized allergen extracts [house dust mite (*Dermatophagoides *sp), cockroach (*Blattella germenica*) and peanut (*Arachis hypogaea*)] from ALK-Abello, Denmark; and some selected fresh fruits (pineapple, mango, banana, papaya, orange, and apple) which are widely consumed in Ghana. The prick-prick procedure, which involves dipping the tip of the skin lancet into a cut portion of the fresh fruit and then pricking the skin with the fruit residue, was used in testing the fruits. Histamine and diluent (ALK-Abello, Denmark) respectively, were used as positive and negative controls. The cut-off for defining a positive reaction to an allergen was an average wheal size of 3 mm (or more) greater than the wheal produced by diluent; and histamine positivity (i.e. wheal size ≥ 3 mm). The results were read 15 minutes after pricking.

#### Anthropometry

Height and weight were measured using a portable free-standing stadiometer and an electronic scale (BS-8001, capacity: 130 kg) respectively. Participants were measured wearing school uniforms and no shoes. In measuring weight, each participant was made to stand still in an upright position on the scale. Weight was recorded to the nearest 0.1 kg whilst height was recorded to the nearest 0.5 cm.

#### Parasitological survey

Participants were asked to provide fresh stool samples in the morning and to submit urine samples between 11:00 and 13:00 hours when maximum shedding of schistosome eggs is known to occur in humans. The Kato-Katz [22] and urine filtration [23] techniques were respectively used in processing stool and urine samples to determine the presence of intestinal helminths and urinary schistosomes. The intensity of infection for intestinal helminths was expressed as number of eggs per gram of faeces whilst the intensity of urinary schistosomes was expressed as number of eggs per 10 ml of urine.

#### Questionnaire survey

Information on potential confounding factors such as; breast-feeding duration, highest educational level and occupation of the person who provided financially for the child (used as proxy measures for socio-economic status), family history of asthma (measured as physician-diagnosed asthma in parents, siblings, grandparents or parents' siblings), source of domestic fuel (i.e. fuel used for cooking at home) and exposure to passive cigarette smoke was obtained through a standardized interviewer-administered questionnaire survey which targeted parents/guardians of the children. The interviews were conducted in the homes of the study participants in English and local Ghanaian languages such as Akan and Ga-Adangme in some cases. Information on participants' date of birth was mainly obtained from their antenatal record cards. Field personnel in this study were trained to conduct measurements and collect data in a standardized way using objective measures.

### Data analysis

Atopy was defined at two levels; atopic (sensitization to at least one tested allergen) and non-atopic (not sensitized to any tested allergen). BMI was calculated as weight (kg) divided by the square of height (m). NutStat, a programme in Epi-Info version 3.3.2 (CDC, Atlanta, Ga., USA), was used in computing the standardized BMI percentiles for age and sex. Based on the CDC growth charts [24], high BMI was defined at the following levels; normal weight: BMI ≥ 5^th ^percentile and < 85^th ^percentile, underweight: BMI < 5^th ^percentile, overweight: BMI ≥ 85^th ^percentile and < 95^th ^percentile and obese: BMI ≥ 95^th ^percentile. For the purpose of this analysis, categories for overweight and obese were merged and referred to as high BMI. Confounding variables were categorized into 3-5 closed-ended levels. To allow all observations to be used in estimating the effect of non-missing observations in the analysis, dummy values were generated for the missing observations of each variable. This was done by creating a missing category for the missing values of each variable. Analysis of variance was used in testing the differences in the mean of height, weight and BMI in the rural and urban sites, whilst adjusting for the effect of age. Logistic regression was used in investigating the association of high BMI and atopy, taking into account confounding factors. A variable was retained in the multivariate model if the p-value corresponding to the unadjusted odds ratio was ≤ 0.10. Sex differentials in urban, respectively rural participants were assessed by inclusion of interaction terms; a p-value of ≤ 0.05 for an interaction term was regarded as evidence of interaction. All analyses were performed using Stata version 9.2 (Stata Corp., College Station, TX).

## Results

### Baseline characteristics

The baseline characteristics of the study population are presented in Table 1. The mean age for the 1482 school children was 10.51 (± 2.29) years and was significantly higher for urban [11.07, (95% CI: 10.90-11.25)] than rural [9.95, (95% CI: 9.80-10.09)] participants. After adjusting for age, the mean height (cm) [urban: 141.96, (95% CI: 141.01-142.90); rural: 131.66, (95% CI: 130.90-132.41)] and weight (kg) [urban: 35.61, (95% CI: 34.80-36.42); rural: 31.03, (95% CI: 30.50-31.56)] differed significantly by residence with participants in the urban site being generally taller and heavier.

### Prevalence of high BMI

**Figure 2:**
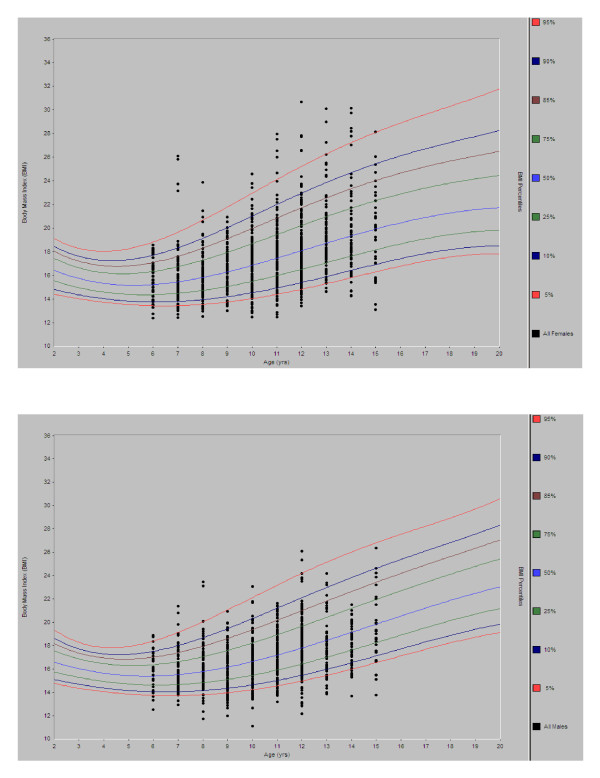
**Distribution of age-standardized BMI by sex in school children aged 6-15 years in the Greater Accra Region of Ghana**.

The prevalence of high BMI, based on standardized BMI-for-age and sex, was 16% (118/740) in the rural site and 10.8% (80/742) in the urban site (p < 0.001). The overall prevalence of high BMI in the study population was 13.4% (198/1482): 13.5% (105/776) in females [urban site: 13.4% (53/395); rural site: 13.6% (52/381)] and 13.2% (93/706) in males [urban site: 7.8% (27/347); rural site: 18.4% (66/359)] (see Figure 2).

### Prevalence of sensitization to individual allergens

Overall, 13.6% [rural: 101/740, urban: 101/742] of the study participants were sensitized to dust mite, 10.8% [rural: 104/740, urban: 56/742, p = 0.000] to cockroach and 1.9% [rural: 15/740, urban: 13/742, p = 0.697] to peanut. The prevalence of sensitization to any of the tested fruits was 3.8% [rural: 24/740, urban: 33/742, p = 0.228]; and of these, pineapple was found to be the most allergenic [1.82% (27/1482)].

**Table 1:** Baseline characteristics of 1,482 school children aged 6-15 years in urban and rural areas of the Greater Accra Region of Ghana

Variable	Urban (%)	Rural (%)	Total p-value**
**All**	**742**	**740**	**1,482**
**Sex**			
Males	347 (46.77)	359 (48.51)	706 (47.64) 0.501
Females	395 (53.23)	381 (51.49)	776 (52.36)
**Age**			
6-8 years	133 (17.92)	191 (25.81)	324 (21.86) 0.000
9-11 years	261 (35.18)	347 (46.89)	608 (41.03)
12-15 years	348 (46.90)	202 (27.30)	550 (37.11)
**Nutritional status indicators**			
Normal weight	568 (76.55)	599 (80.95)	1,167 (78.74) 0.000
Underweight	94 (12.67)	23 (3.11)	117 (7.89)
High BMI	80 (10.78)	118 (15.95)	198 (13.36)
**Atopy**			
Non-atopic	610 (82.21)	554 (74.86)	1164 (78.54) 0.001
Atopic	132 (17.79)	186 (25.14)	318 (21.46)
***S. haematobium *infection**			
Not infected	714 (97.28)	630 (90.00)	1,344 (93.72) 0.000
Infected	20 (2.72)	70 (10.00)	90 (6.28)
**Intestinal parasites infection**			
Not infected	659 (97.34)	434 (57.81)	1, 093 (82.99) 0.000
Hookworm	13 (1.92)	76 (11.88)	89 (6.76)
*Trichuris sp*	2 (0.30)	16 (2.50)	18 (1.37)
*Ascaris sp*	0 (0.00)	93 (14.53)	93 (7.06)
**^Ж^**Co-infected	3 (0.44)	21 (3.28)	24 (1.82)
**Passive smoking**			
Non-exposed	398 (66.22)	218 (69.43)	616 (67.32) 0.327
Exposed	203 (33.78)	96 (30.57)	299 (32.68)
**Source of domestic fuel**			
Liquefied Petroleum Gas (LPG)	368 (61.13)	5 (1.59)	373 (40.68) 0.000
Charcoal	184 (30.56)	111 (35.24)	295 (32.17)
Firewood	50 (8.31)	199 (63.17)	249 (27.15)
**Family history of asthma**			
No	352 (59.26)	174 (55.24)	526 (57.87) 0.338
Yes	170 (28.62)	105 (33.33)	275 (30.25)
No idea	72 (12.12)	36 (11.43)	108 (11.88)
**Exclusive breast feeding**			
None	74 (18.23)	48 (10.39)	122 (14.06.) 0.002
1-6 months	280 (68.97)	360 (77.92)	640 (73.73)
6-12 months	52 (12.81)	54 (11.69)	106 (12.21)
**Educational level of parent**			
No formal education	124 (27.43)	148 (30.14)	272 (28.84) 0.011
7-9 years education	178 (39.38)	165 (33.60)	343 (36.37)
10-15 years education	68 (15.04)	108 (22.00)	176 (18.66)
Tertiary education	82 (18.14)	70 (14.26)	152 (16.12)
**Occupation of parent**			
Unemployed	16 (3.56)	14 (2.86)	30 (3.20) 0.542
Trader	76 (16.93)	139 (28.43)	215 (22.92)
Farmer	95 (21.16)	83 (16.97)	178 (18.98)
Public servant	168 (37.42)	169 (34.56)	337 (35.93)
Business	94 (20.94)	84 (17.18)	178 (18.98)

^Ж ^Co-infection: infected with two or more intestinal helminthes

** Comparison between urban and rural areas. Totals may differ due to missing values of some variables.

### Prevalence of atopy

25.1% [186/740] of the rural participants and 17.8% [132/742] of the urban participants were found to be atopic; with the overall prevalence of atopy being 21.5% (318/1,482). A higher prevalence of atopy was observed in males [rural: 29.0% (104/359); urban: 22.8% (79/347)] than females [rural: 21.5% (82/381); urban: 13.4% (53/395)]. The prevalence of atopy in the study population also increased considerably with age [6-8 years: 18.8% (61/324); 9-11 years: 20.7% (126/608); 12-15 years: 23.8% (131/550)]; however, this increase was more profound in the urban site (data not shown).

**Table 2:** Factors associated with atopy in 1,482 school children aged 6-15 years living in urban and rural areas in the Greater Accra Region of Ghana

		UNIVARIATE ANALYSIS			MULTIVARIATE ANALYSIS**		
**Variable **	Atopy (%)*	**Combined** **OR (95% CI)**	**Urban** **OR (95% CI)**	**Rural** **OR (95% CI)**	**Combined** **OR (95% CI)**	**Urban** **OR (95% CI)**	**Rural** **OR (95% CI)**

**Sex**							
Female	135 (17.4)	1	1	1	1	1	1
Male	183 (25.9)	1.66 (1.29-2.13)	1.90 (1.30-2.79)	1.49 (1.06-2.08)	1.63 (1.26-2.11)	2.10 (1.41-3.13)	1.45 (1.03-2.04)
**Age**							
6-8 years	61 (18.8)	1	1	1	1	1	1
9-11 years	126 (20.7)	1.13 (0.80-1.58)	1.12 (0.61-2.05)	1.15 (0.76-1.75)	1.14 (0.80-1.63)	1.19 (0.63-2.24)	1.18 (0.76-1.82)
12-15 years	131 (23.8)	1.35 (0.96-1.90)	1.76 (1.00-3.07)	1.32 (0.83-2.09)	1.36 (0.95-1.96)	1.89 (1.05-3.42)	1.28 (0.79-2.07)
**Nutritional status indicators**							
Normal weight	254 (21.8)	1	1	1	1	1	1
Underweight	254 (21.8)	0.61 (0.36-1.04)	0.58 (0.30-1.12)	1.08 (0.42-2.78)	0.57 (0.33-0.99)	0.52 (0.26-1.02)	1.07 (0.40-2.83)
High BMI 47 (23.7)	254 (21.8)	1.12 (0.78-1.60)	1.00 (0.55-1.83)	1.13 (0.73-1.77)	1.17 (0.81-1.69)	1.23 (0.66-2.29)	1.17 (0.73-1.87)
**Passive smoking**							
Non-exposed	125 (20.3)	1	1	1			
Exposed	61 (20.4)	1.00 (0.71-1.42)	0.78 (0.50-1.23)	1.54 (0.89-2.66)			
**Family history of asthma**							
No 94 (17.9)	61 (20.4)	1	1	1	1	1	1
Yes	70 (25.5)	1.57 (1.10-2.23)	1.58 (1.01-2.47)	1.52 (0.86-2.67)	1.56 (1.09-2.24)	1.54 (0.97-2.46)	1.41 (0.79-2.53)
No idea	20 (18.5)	1.04 (0.61-1.78)	0.80 (0.39-1.65)	1.53 (0.67-3.46)	0.99 (0.58-1.72)	0.68 (0.32-1.43)	1.56 (0.68-3.60)
**Occupational status of parent**							
Unemployed	9 (30.0)	1	1	1	1	1	1
Employed	183 (20.2)	0.59 (0.27-1.31)	0.33 (0.12-0.93)	1.13 (0.31-4.13)	0.65 (0.29-1.47)	0.33 (0.11-1.00)	1.26 (0.32-4.95)

**Source of domestic fuel**							
LPG***	77 (20.6)	1	1	1			
Charcoal	50 (16.9)	0.78 (0.53-1.16)	0.75 (0.47-1.19)	0.88 (0.09-8.29)			
Firewood	59 (23.7)	1.19 (0.81-1.75)	0.52 (0.22-1.28)	1.45 (0.16-13.29)			
**Exclusive breast feeding**							
None	22 (18.0)	1	1	1			
1-6 months	138 (21.6)	1.25 (0.76-2.06)	1.29 (0.62-2.70)	1.01 (0.51-2.03)			
6-12 months	17 (16.0)	0.87 (0.43-1.74)	1.34 (0.50-3.57)	0.52 (0.19-1.41)			
**Educational level of parent**							
No formal education	57 (21.0)	1	1	1	1	1	1
7-9 years education	67 (19.3)	0.92 (0.62-1.36)	0.76 (0.43-1.37)	1.09 (0.64-1.87)	0.92 (0.61-1.39)	0.68 (0.37-1.26)	0.97 (0.56-1.70)
10-15 years education	35 (19.9)	0.94 (0.58-1.50)	0.43 (0.18-1.06)	1.32 (0.74-2.37)	0.98 (0.60-1.59)	0.44 (0.17-1.10)	1.24 (0.68-2.26)
Tertiary education	34 (22.4)	1.09 (0.67-1.76)	0.78 (0.38-1.59)	1.51 (0.79-2.90)	1.07 (0.65-1.75)	0.67 (0.32-1.41)	1.36 (0.70-2.65)
**Intestinal parasite infection**							
Not infected	218 (20.0)	1	1	1	1		1
Hookworm	26 (29.2)	1.66 (1.02-2.68)	1.39 (0.38-5.13)	1.43 (0.84-2.45)	1.39 (0.84-2.83)	-	1.34 (0.77-2.33)
*Trichuris* sp	5 (27.8)	1.54 (0.54-4.28)	-	1.50 (0.51-4.41)	1.53 (0.53-4.39)	-	1.50 (0.50-4.50)
*Ascaris* sp	19 (20.4)	1.03 (0.61-1.74)	-	0.85 (0.49-1.47)	1.00 (0.57-1.74)	-	0.79 (0.45-1.38)
Co-infected	9 (37.5)	2.41 (1.04-5.58)	-	2.47 (1.01-6.04)	2.13 (0.90-5.04)	-	2.23 (0.90-5.58)
***S. haematobium *infection**							
No	290 (21.6)	1	1	1			
Yes	20 (22.2)	1.04 (0.62-1.74)	0.81 (0.23-2.80)	0.93 (0.52-1.65)			

* Totals for atopy may differ due to missing values of some variables.

** Adjusted for all other variables in the analysis. For inclusion in the multivariate model, a variable's p-value for the unadjusted odds ratio had to be ≤ 0.10

*** LPG: Liquefied Petroleum Gas

### Association between high BMI and atopy

Details of the relationship between the study parameters and atopy are given in Table 2. Using normal weight as our reference, high BMI and atopy were not associated in the study population; whereas underweight was found to be inversely associated with atopy after multivariate adjustment [OR: 0.57, 95% CI: 0.33-0.99; p = 0.045]. However, this effect was only significant in males [OR: 0.49, 95% CI: 0.25-0.98; p = 0.045].

### Association of other study parameters with atopy

In both the rural [OR: 1.49, 95% CI: 1.06-2.08] and urban [OR: 1.90, 95% CI: 1.30-2.79] sites, males had an increased likelihood of being atopic compared to females. The likelihood of atopy was also increased in older urban children [OR: 1.76, 95% CI: 1.00-3.07] and those with a family history of asthma [OR: 1.57, 95% CI: 1.10-2.23]. No regular trend was observed across the various levels of parent's occupation and atopy. However, on recoding parent's occupation into a binary variable (unemployed and employed), the likelihood of atopy was observed to be decreased [OR: 0.33, 95% CI: 0.12-0.93] in urban participants with employed parent(s). Being co-infected with two or more intestinal parasites in the rural site was associated with increased odds of atopy by more than two-folds [OR: 2.47, 95% CI: 1.01-6.04].

Stratification by sex for rural and urban participants showed decreased likelihood of atopy for urban females with employed parents (OR: 0.22, 95% CI: 0.05-0.99) and increased likelihood of atopy for urban males aged 12-15 years (OR: 2.18, 95% CI: 1.02-4.66). Rural females were significantly more likely to be atopic if intestinal parasite co-infection occurred (OR:4.69; 95% CI 1.30-16.93) and if their parents attended school for 7-9 years (OR: 2.68, 95% CI: 1.17-6.15) or had tertiary education (OR: 4.89, 95% CI 1.73-13.80). Inclusion of a sex/educational attainment interaction term in the logistic regression model showed significant interaction (p = 0.003), whereas interaction terms for sex and age, parent's educational attainment or intestinal parasite co-infection were not significant.

After multivariate adjustment, the following variables remained significantly associated with atopy: male sex [rural: OR: 1.45, 95% CI: 1.03-2.04; urban: OR: 2.10, 95% CI: 1.41-3.13], family history of asthma [OR: 1.56, 95% CI: 1.09-2.24] and older age (i.e. 12-15 years) in the urban site [OR: 1.89, 95% CI: 1.05-3.42].

## Discussion

High BMI in this population of Ghanaian school children aged 6 to 15 years was not associated with atopy in both the rural and urban sites. However, underweight children were found to have a reduced likelihood of atopy. Other factors such as sex, older age, family history of asthma, occupational status of parents, educational level of parents and co-infection with intestinal parasites were associated with atopy in univariate analysis; whilst male sex, older age, and family history of asthma remained the significant predictors of atopy after multivariate adjustment. In the rural site a higher prevalence of high BMI and atopy were observed than in the urban site in this study.

In line with previous studies, the urban participants in our study were considerably taller and heavier than their rural counterparts [3,18]. Compared to studies in other parts of Ghana [3], the anthropometric parameters for this study population are slightly lower; but this could be attributed to the lower mean age of our study population.

Previous studies have often shown a link between lower levels of physical activity and high BMI [21]. However, children in most rural settings in Africa are normally expected to participate in the household chores which may include working on the farm, carrying water from the riverside or public taps; and in coastal communities such as the rural site in this study, accompany their fathers on fishing expeditions (which involves the paddling of manual canoes). Despite this, a higher prevalence of high BMI was recorded in our rural participants in this study. A possible explanation for this observation could be that early life factors such as malnutrition had increased the risk of central adiposity in the rural participants. Available records show that the prevalence of underweight has consistently been high among children under the age of five in rural areas in Ghana [17]. Studies have shown that malnutrition occurring in early life compromises linear growth, with subsequent availability of food potentially increasing the risk of central adiposity due to the increase in body weight but not height [25].

Previous studies in Ghana [26] and other parts of Africa [4] have generally reported a higher prevalence of sensitization to dust mite and cockroach allergens as compared to allergens from grass pollen and domestic animals such as cat and dog. The other allergens which were included in our panel (peanut and fruits), were used to study the levels of sensitization to these food items which constitute the diet of many Ghanaians. Hence, we are convinced that our panel of allergens was able to detect the majority of atopic children in the study population. In contrast to previous studies [3,13,18,27] the current study found a higher prevalence of atopy in the rural site. A possible explanation for this observation could be the significantly higher levels of cockroach sensitivity which was recorded in the rural site in this study.

The exposure and sensitivity to cockroach allergens within countries has been shown to exhibit a geographical variation [28] and the prevalence of cockroach sensitivity has generally been reported to be high among low socio-economic populations in urban areas [29]. Like most previous studies [30], diagnosis of cockroach sensitivity in this study was based on sensitization to *Blattella germenica*. However, other species of cockroaches such as *Periplaneta americana *and *Blatta orientalis *are common in Ghana; though there are no available data on their distribution and abundance. Since sensitization to cockroach allergen is dependent on exposure and most cockroach allergens have been reported to be species-specific [28], it is likely that *Blattella germenica *is more prevalent in the rural than the urban site in this study. Further studies are however required to investigate this.

The association between high BMI and atopy has been explored in cross-sectional [13,30-32], case control [19,33] and cohort [34] studies with often inconsistent findings. Positive associations have been reported in cross-sectional surveys on Taiwanese teenagers [13] Caucasian Australian children [32] and American children [31], whereas a study in Chinese school children did not find an association between obesity and atopy [35]. In this study, high BMI was not associated with atopy, whereas a significant inverse association was observed between underweight and atopy in males. This supports studies which have reported that skin test sensitivity is low in thin children [19] but contradicts the findings of a similar survey in Belgium [31] where underweight girls were found to have an increased risk of atopy. Our finding could partly be explained by previous studies in animal models [36] which showed that the production of primary signals for atopy such as interleukin-4 is down-regulated when nutritional intake is reduced. This is mainly because levels of circulating leptin, interleukin-6, interleukin-8, C-reactive protein, plasminogen activator inhibitor-1 and haptoglobin, which are all markers of inflammation, are considerably decreased in underweight individuals [15] High levels of these markers collectively contribute to the skewing of the immune system towards a T-helper lymphocyte type 2 (Th2) cytokine profile which results in an increased risk of atopy [15]. Hence, it is possible that reduced levels of these markers may increase immune tolerance to antigens in underweight individuals. Reasons why this inverse association was only observed in males are not immediately known but could be due to the higher proportion of underweight males than females (57.3% vs. 42.7%) in this study. Previous studies have reported positive associations between underweight and asthma in boys [37,38]. However, these studies did not verify if reported asthma was due to atopic or non atopic mechanisms. In this study, no information was collected on asthma symptoms, thus not allowing us to comment on implications with regard to asthma development.

The higher prevalence of atopy which was observed among males in this study is in line with the findings of other studies [13]. Family history of asthma has been shown to increase susceptibility to atopy whilst the risk of atopy increases with the age of children [39]. As exposure normally precedes sensitization, it is possible that the degree of allergen exposure increases with age. Hence, the more profound increase in atopy with age in the urban site could be attributed to the higher mean age of the urban participants in this study.

Over the years, most studies [3] have consistently shown a link between higher socio-economic status (SES) and atopy. Some of the plausible explanations which have been offered for this association include better nutritional status at higher SES in turn influencing the function of T-lymphocyte cells. SES may influence the production of IgE through T-helper lymphocyte cell function [39]. In this study, the employment status and educational levels of parents were used as proxy measures of SES and the observed association between parental educational level and atopy is consistent with some previous studies [13]. This notwithstanding, reasons for the observed inverse association with parent's employment status are quite unclear but could be due to confounding by factors such as diet which was not measured in this study.

Early studies on allergy among helminth infected populations in parts of Africa often reported that the levels of allergic sensitization in such populations were low compared to non-infected populations. Hence, it was suggested that Th2-inducing infections might actually protect against atopy [5,6]. More recent studies have shown that the role of helminth infections in the development of allergy may not always be protective but could be influenced by the intensity or chronicity of infections [6]. For instance, children with wealthy parents often tend to exhibit higher levels of atopy and harbour lower helminth burdens whilst the converse applies to children with poor parents. Therefore, it has been proposed that SES may influence the burden of helminth infections through nutritional status which consequently influences T-helper lymphocyte cell function [40]. Hence, light acute infections may enhance atopy whilst heavy and chronic infections may inhibit it [6,41]. In this study, we found that infection with two or more intestinal helminths at the same time in an individual was associated with an increased likelihood of atopy in the rural site. It is possible that the combined effect produced by different species of intestinal helminths when they occur together might be different from when they occur individually.

With the rapid increase in rural-urban migration in sub-Saharan African countries and the rise in childhood allergy cases in urban areas, it was important to determine if high body weight which is increasingly becoming common in developing countries influences the development of atopy. To our knowledge, this is the first study which has examined the relation between high BMI on atopy in rural and urban settings in West Africa. Field personnel in this study were trained to collect data in a standardized way using objective measures; hence, the potential for misclassification of atopy and BMI was kept to a minimum. Though there were missing observations of some variables, our analyses showed that the missing observations did not significantly influence the findings of the study. Regardless of this, the results of this study should be interpreted in the context of a response rate of 59% and the potential for selection bias. Whereas parents from lower socio-economic levels may regard research as an easier means of getting free access to health care, the converse applies to parents in higher socio-economic levels. Hence, our study population in the urban site consists mainly of participants from public and middle-high private schools but not the very high class schools. It is therefore possible that the observed prevalence of high BMI and atopy in the urban site does not reflect the true prevalence of these conditions and that the associations observed between these two might be biased.

The use of the CDC growth reference charts in classifying nutritional status in this study is another potential source of bias. Aside from limitations it shares with other available growth reference charts [42], the CDC charts had American children as its reference population [43]. Hence, its application to children in an African country may not provide accurate estimates.

## Conclusion

In our study population of Ghanaian school children, high BMI was not associated with atopy, whereas sex, older age and family history of asthma were. These findings do not support obesity as an important risk factor for atopy status in this population.

## List of abbreviations used

BMI: Body Mass Index; 95% CI: 95% Confidence Interval; IgE: immunoglobulin E antibodies; OR: Odds Ratio; SES: Socio-economic status; SPT: Skin Prick Testing.

## Competing interests

The authors declare that they have no competing interests.

## Authors' contributions

IAL designed the study, was involved in data collection and management, conducted the statistical data analysis and drafted the manuscript. KKG was involved in the design of the study, guided statistical analysis and manuscript writing. ASA was involved in data collection and management and reviewed the manuscript. BOB and MDW were involved in project implementation and review of the manuscript. MY was responsible for the overall project coordination of the European Union funded project of which this study formed a part and reviewed the manuscript. DAB was involved in the design of the study, the overall project implementation and co-ordination and reviewed the manuscript. All authors have read and approved the final manuscript.

## Pre-publication history

The pre-publication history for this paper can be accessed here:

http://www.biomedcentral.com/1471-2458/11/469/prepub
